# Cytokine and chemokine profiles in pulmonary tuberculosis with pre-diabetes

**DOI:** 10.3389/fimmu.2024.1447161

**Published:** 2024-08-29

**Authors:** Anuradha Rajamanickam, Sanaadhan P. Kothandaraman, Nathella Pavan Kumar, Vijay Viswanathan, Sivakumar Shanmugam, Syed Hissar, Sujatha Nott, Hardy Kornfeld, Subash Babu

**Affiliations:** ^1^ International Center for Excellence in Research, National Institute of Allergy and Infectious Diseases (NIAID), Chennai, India; ^2^ Stanley Medical College, Chennai, India; ^3^ Department of Immunology, Indian Council of Medical Research (ICMR)-National Institute for Research in Tuberculosis, Chennai, India; ^4^ Prof. M. Viswanathan Diabetes Research Center, Chennai, India; ^5^ Department of Bacteriology, Indian Council of Medical Research (ICMR)-National Institute for Research in Tuberculosis, Chennai, India; ^6^ Department of Clinical Research, Indian Council of Medical Research (ICMR)-National Institute for Research in Tuberculosis, Chennai, India; ^7^ Infectious Diseases, Dignity Health, Chandler, AZ, United States; ^8^ Department of Medicine, University of Massachusetts Medical School (UMass) Chan Medical School, Worcester, MA, United States; ^9^ Laboratory of Parasitic Diseases (LPD), National Institute of Allergy and Infectious Diseases (NIAID), National Institutes of Health (NIH), Rockville, MD, United States

**Keywords:** pre-diabetes, tuberculosis, cytokines, chemokines, metabolism, immunity

## Abstract

**Introduction:**

Tuberculosis (TB) remains a significant health concern in India, and its complexity is exacerbated by the rising occurrence of non-communicable diseases such as diabetes mellitus (DM). Recognizing that DM is a risk factor for active TB, the emerging comorbidity of TB and PDM (TB-PDM) presents a particular challenge. Our study focused on the impact of PDM on cytokine and chemokine profiles in patients with pulmonary tuberculosis TB) who also have PDM.

**Materials and methods:**

We measured and compared the cytokine (GM-CSF, IFN-γ, IL-1α/IL-1F1, IL-1β/IL-1F2, IL-2, IL-4, IL-5, IL-6, IL-10, IL-12p70, IL-13, IL-17/IL-17A, IL-18/IL-1F4, TNF-α) and chemokine (CCL1, CCL2, CCL3, CCL4, CCL11, CXCL1, CXCL2, CXCL9, CXCL10, and CXCL11) levels in plasma samples of TB-PDM, only TB or only PDM using multiplex assay.

**Results:**

We observed that PDM was linked to higher mycobacterial loads in TB. Patients with coexisting TB and PDM showed elevated levels of various cytokines (including IFNγ, TNFα, IL-2, IL-17, IL-1α, IL-1β, IL-6, IL-12, IL-18, and GM-CSF) and chemokines (such as CCL1, CCL2, CCL3, CCL4, CCL11, CXCL1, CXCL9, CXCL10, and CXCL11). Additionally, cytokines such as IL-18 and GM-CSF, along with the chemokine CCL11, were closely linked to levels of glycated hemoglobin (HbA1c), hinting at an interaction between glycemic control and immune response in TB patients with PDM.

**Conclusion:**

Our results highlight the complex interplay between metabolic disturbances, immune responses, and TB pathology in the context of PDM, particularly highlighting the impact of changes in HbA1c levels. This emphasizes the need for specialized approaches to manage and treat TB-PDM comorbidity.

## 1 Introduction

Tuberculosis (TB) remains a leading cause of death worldwide. Globally, million people were estimated to be newly diagnosed with TB with an estimated death due to TB being 1.3 million in 2022 (1). The intersection of TB and type 2 diabetes (T2D) significantly worsens health outcomes, increasing the risk of drug-resistant TB strains and elevating relapse and mortality rates during treatment (1, 2). In India, approximately 26% of TB patients also suffer from diabetes, highlighting a critical public health issue (3, 4).

Pre-diabetes (PDM), a condition of intermediate hyperglycemia characterized by impaired fasting glucose (IFG) or impaired glucose tolerance (IGT), affects nearly 298 million people worldwide—a figure projected to rise to 414 million by 2045 (5). PDM not only increases the likelihood of progressing to diabetes but also amplifies the risk of developing TB. This is attributed to an impaired immune response which is further compromised in the presence of active TB, potentially exacerbating dysglycemia (6–8).

Further, exacerbating the complexity, recent studies suggest that the immune systems of individuals with PDM are dysregulated, showing enhanced levels of pro-inflammatory cytokines including Type 1 and Type 17 cytokines and others like IL-1β, IFNβ, and GM-CSF, which are crucial in the pathogenesis of TB (9, 10). This cytokine profile is similar to that of patients with TB and diabetes, indicating a similar alteration in immune function that could exacerbate TB progression (3, 9, 11–21). Moreover, PDM has been shown to be associated with unfavorable treatment outcomes in TB (9, 14, 22, 23).

Given the increasing prevalence of PDM and its potential to progress to diabetes, understanding how PDM influences TB pathogenesis is crucial. This study aims to explore the impact of PDM on the cytokine and chemokine responses in patients newly diagnosed with active pulmonary tuberculosis (TB). By comparing cytokine levels in TB patients with and without PDM, we seek to unravel how even modest disruptions in glycemic control could influence the inflammatory milieu in TB, potentially informing better management strategies for this dual burden of disease.

## 2 Methods and materials

### 2.1 Ethics statement

This study was approved by the Ethics Committees of the Prof. M. Viswanathan Diabetes Research Center (ECR/51/INST/TN/2013/MVDRC/01) and NIRT (NIRT-INo:2014004). Informed written consent was obtained from all participants. All the methods were performed in accordance with institutional ethical committee guidelines. The study participants were recruited from the Effect of Diabetes in Tuberculosis Severity protocol conducted under the RePORT (Regional Prospective Observational Research for Tuberculosis) India consortium.

### 2.2 Study population

Individuals newly diagnosed with smear and culture-positive pulmonary TB, both with and without PDM, were enrolled between 2015 – 2018 in Chennai. This study analyzed baseline plasma samples from a cohort of 66 participants divided into three groups: 22 individuals co-positive for PDM and TB, 22 positive for TB and normoglycemic (NDM), and 22 PDM who were TB-negative. TB was diagnosed based on sputum smear and culture positivity, employing Ziehl-Nielsen smear microscopy and culture grading. In brief, before beginning anti-TB medication, all patients had three sputum samples (spot-morning-spot) taken after being instructed and shown how to provide high-quality sputum. Using the Ziehl-Neelsen (ZL) method, all of the sputum samples were stained for acid-fast bacilli (AFB). Individuals with pulmonary TB were diagnosed by positive solid cultures in Lowenstein–Jensen medium and were classified as 1+ (10–100 colonies), 2+ (>100–200 colonies) and 3+ (>200 colonies). Chest radiographs on enrollment were graded by two blinded readers using a validated severity score based on the percent area of lung involved with TB disease and the presence or absence of cavities. Smear grades were used to determine bacterial burdens and classified as 1+ (10–99 AFB in 100 fields), 2+ (1–10 AFB in each field) and 3+ (more than 10 AFB in each field) based on World Health Organization guidelines and according to the NTEP national laboratory guidelines (24). All standard test methods for smear, culture (solid and liquid), and Xpert MTB/RIF shall be carried out. Chest X-rays were used to determine cavitary disease and unilateral versus bilateral involvement. PDM was defined by a Glycated Hemoglobin (HbA1c) level of 5.7% to 6.4%, according to the American Diabetes Association criteria. PDM individuals were asymptomatic with normal chest X-rays and Quantiferon TB-Gold. Comprehensive assessments including anthropometric measurements (e.g., BMI) and biochemical parameters (e.g., HbA1c, random blood glucose, total cholesterol, serum triglycerides, HDL, and LDL cholesterol) were conducted. Additional clinical features, such as the presence of cavitary lesions and bilateral lung disease, were recorded.

### 2.3 Multiplex assay methodology

The levels of cytokines and chemokines were measured using the Bio-Rad, MAGPIX multiplex system (Bio-Rad, MAGPIX multiplex reader, xPONENT 4.2 acquisition and Bio-plex manager 6.1 software). Luminex Human Cytokines Magnetic Assay kit (R & D systems, USA) In brief, samples of plasma were purified and stored frozen at −80 °C prior to Luminex assays. The samples were thawed to room temperature and following the manufacturer’s recommendations, the assay was performed to measure the levels of cytokines and chemokines. The lowest detection limits for cytokines were as follows: GM-CSF, 18.4 pg/mL; IFN-γ, 5.7 pg/mL; IL-1α/IL-1F1, 10.6 pg/mL; IL-1β/IL-1F2, 3.5 pg/mL; IL-2, 3.6 pg/mL; IL-4, 1.1 pg/mL; IL-5, 6.2 pg/mL; IL-6, 9.0 pg/mL; IL-10, 32.2 pg/mL; IL-12p70, 18.5 pg/mL; IL-13, 31.8 pg/mL; IL-17/IL-17A, 9 pg/mL; IL-18/IL-1F4, 2.5 pg/mL; TNF-α, 12.4 pg/mL and chemokines like CCL1, 1.57 pg/mL; CCL2, 31.8 pg/mL; CCL3, 90.9 pg/mL; CCL4, 103.8 pg/mL; CCL11, 21.6 pg/mL; CXCL1, 49.2 pg/mL; CXCL2, 49.2 pg/mL; CXCL9, 600.6 pg/mL; CXCL10, 2.88 pg/mL and CXCL11, 21.6 pg/mL.

### 2.4 Statistical analysis

Geometric means (GM) were calculated to describe the central tendency of data. Group comparisons among the TB-PDM, TB, and PDM were made using the Kruskal-Wallis test, with Dunn’s multiple comparisons test applied to identify statistically significant differences. The Spearman rank correlation coefficient was used to assess correlations between variables. The cytokine and chemokine levels were correlated with HbA1c levels. The linear regression analysis was performed between the levels of cytokines and HbA1c, RBG and total cholestrol. Data analysis was performed using GraphPad Prism version 10.2.3 and JMP version 17.0.0.

## 3 Results

### 3.1 Characteristics of the study population

The demographic and biochemical baseline characteristics of the study population are presented in **Table 1**. Individuals with TB-PDM exhibited significantly higher levels of HbA1c (TB-PDM; Geomean (GM) 5.92, IQR 5.9–6.12 vs. TB, GM 5.6, IQR 4.12–5.35; PDM, GM 5.3, IQR 5.2–5.4; p = 0.0286), random blood glucose (RBG) (TB-PDM; GM 101, IQR 80–148 vs. TB, GM 86, IQR 74–116; PDM, GM 85, IQR 68–115; p = 0.0160), and total cholesterol (TB-PDM; GM 168, IQR 145–198 vs. TB, GM 146, IQR 128–190; PDM, GM 131, IQR 105–140; p = 0.0386) compared to those with TB alone and PDM alone. No significant differences were observed in age, sex, BMI, serum triglycerides, high-density lipoprotein cholesterol (HDL), or low-density lipoprotein cholesterol (LDL) among the TB-PDM, TB, and PDM groups. As detailed in **Table 2**, the subgroup with TB-PDM demonstrated significantly higher bacterial loads than the TB-only group, with mean scores of 3+ in acid-fast bacillus (AFB) staining and 4+ in culture results (p = 0.035 and p = 0.001, respectively). The prevalence of bilateral pulmonary involvement and occurrence of pulmonary cavities were comparable across the groups, with no statistically significant differences noted.

**Table 1:** Demographics and biochemistry profile of TB-PDM and TB and PDM individuals.

Parameter	TB -PDM n=22	TB n=22	PDM n=22	p value
**Age**	41 (18-65)	39.5 (20-65)	41.5 (22-65)	0.5647
**Gender M/F**	10/12	12/10	12/10	0.6821
**BMI (kg/m^2^)**	22.5 (16.4 – 24.5)	20.4 (14.6 – 22.3)	24.5 (15.5 – 30.10)	0.4876
**Smear Grade: 0/1+/2+/3+**	0/12/4/6	0/9/10/3	NA	
**Cavitary Disease (Y/N)**	6/16	5/17	NA	
**Lung Lesions (Unilateral/Bilateral)**	15/7	16/6	NA	
Biochemical Parameters				
**HbA1c (%)**	5.92 (5.71–6.44)	5.6 (4.04–5.65)	5.3 (5.2– 5.4)	0.0286
**RBG (mg/dl)**	101 (78– 156)	86 (70–120)	85 (66–118)	0.0160
**Total cholesterol (mg/dl)**	168 (142–202)	146 (124–195)	131 (102-148)	0.0386
**Serum triglycerides (mg/dl)**	102 (64–424)	98 (56–384)	141 (50-179)	0.6382
**HDL (ml/dl)**	37 (29–59)	45 (25–66)	52.8 (30-96)	0.5926
**LDL (ml/dl)**	104 (44–180)	96 (52–146)	83. 5 (60-195)	0.6366

The values represent the geometric mean and the range (except for age where the median and the range) are shown.

Smear grades were used to determine bacterial burdens and classified as 1+ (10–99 AFB in 100 fields), 2+ (1–10 AFB in each field) and 3+ (more than 10 AFB in each field) based on World Health Organization guidelines.

Unilateral Lung Lesions: These are lesions that affect only one lung or one side of the chest. They may appear as localized abnormalities on imaging such as X-rays.

Bilateral Lung Lesions: These lesions affect both lungs simultaneously. They can manifest as widespread abnormalities throughout both lungs or as multiple discrete lesions scattered across both lung fields.

**Table 2:** Clinical profile of TB-PDM Vs TB individuals.

	TB-PDM (n = 22)	TB (n = 22)	p-value
AFB			
**1+**	12	9	0.035
**2+**	4	10	
**3+**	6	3	
Culture			
**1+**	14	13	0.001
**2+**	1	9	
**3+**	7	0	
Cavity			
**Yes**	6	5	0.728
**No**	16	17	
Bilateral			
**Yes**	7	6	0.819
**No**	15	16	

AFB smear result with respect to the number of M. tuberculosis culture-positive specimens from three consecutive days of sputum samples.

Smear grades were used to determine bacterial burdens and classified as 1+ (10–99 AFB in 100 fields), 2+ (1–10 AFB in each field) and 3+ (more than 10 AFB in each field) based on World Health Organization guidelines. According to the NTEP national laboratory guidelines.

Chest X-rays were used to determine cavitary disease and unilateral versus bilateral involvement.

### 3.2 TB-PDM is linked to higher systemic levels of type 1 and type 17 cytokines

**Figure 1:**
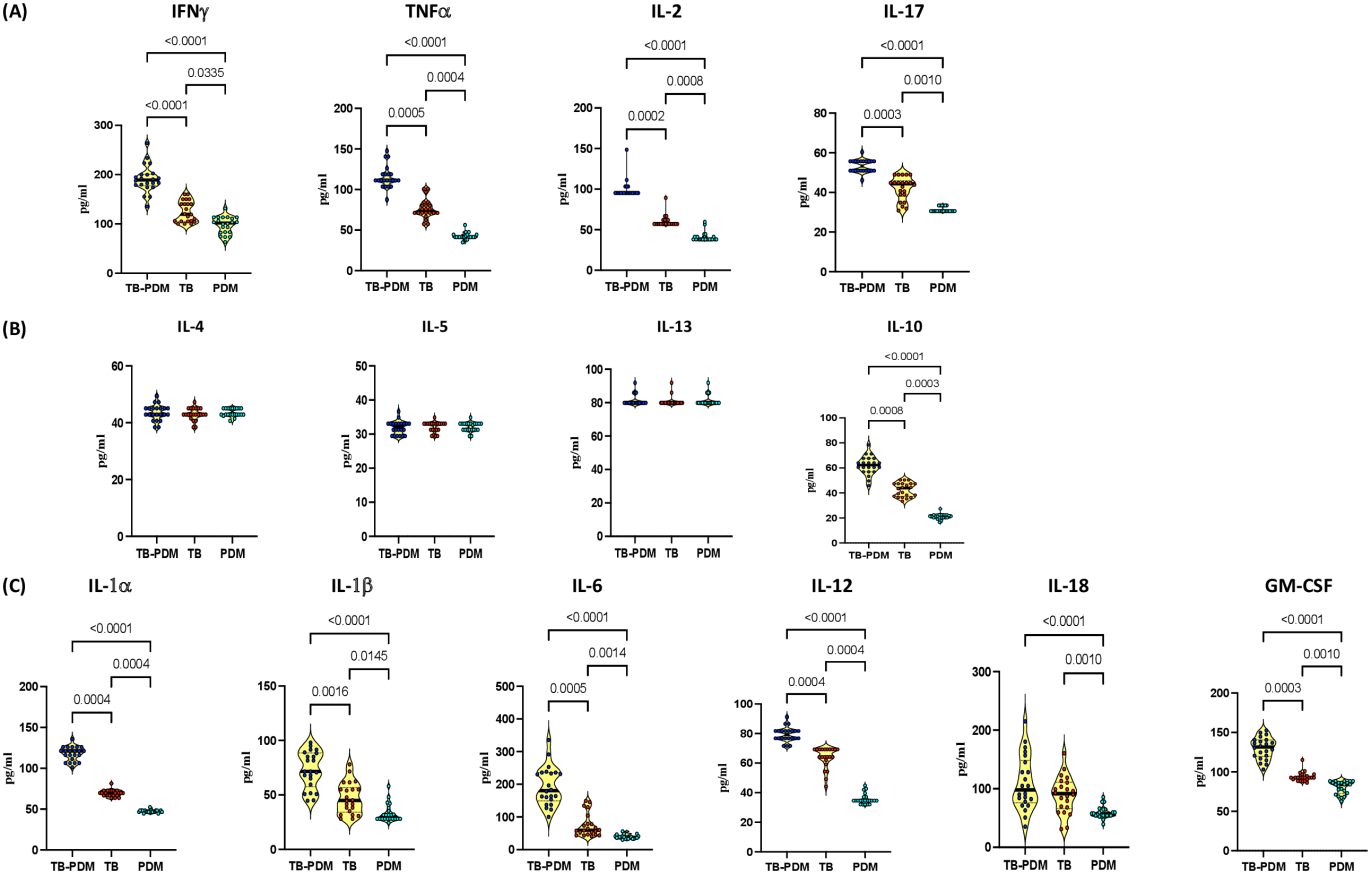
Elevated Levels of Type 1 and Type 17 Cytokines in TB-PDM. Plasma levels of **(A)** Type 1 (IFNγ, TNFα, IL-2) and Type 17 (IL-17) cytokines, **(B)** Type 2 (IL-4, IL-5, IL-13), and regulatory (IL-10) cytokines, **(C)** and other pro-inflammatory cytokines (IL-1α, IL-1β, IL-6, IL-12, IL-18, and GM-CSF) were measured via Bio-Rad, MAGPIX multiplex system in individuals with TB-PDM (n=22), PDM (n=22), and TB (n=22). Data are presented as violin plots, with each circle representing an individual (Navy Blue – TB-PDM; Maroon – TB; Light Blue – PDM). p-values were calculated using the Kruskal-Wallis test with Dunn’s multiple corrections.

To explore the influence of PDM on the levels of type 1 and type 17 cytokines in individuals with active TB, we analyzed the circulating concentrations of these cytokines. Specifically, we measured type 1 cytokines (IFN-γ, TNF-α, and IL-2) and the type 17 cytokine IL-17 in groups with TB-PDM, TB alone, and PDM alone. As illustrated in **Figure 1A**, the levels of type 1 cytokines were substantially elevated in the TB-PDM group (IFN-γ: TB-PDM-median, 189.2 pg/ml; IQR, 177.6-200.5 pg/ml Vs TB, median 119.5 pg/ml; IQR, 105.0-142.5 pg/ml Vs PDM, median 101.8 pg/ml; IQR, 83.08-113.3 pg/ml: p<0.0001) compared to those with only TB or PDM. Similar patterns were observed for TNF-α (TB-PDM-median, 111.2 pg/ml; IQR, 109.3-118.8 pg/ml Vs TB, median 73.50 pg/ml; IQR, 71.28-81.15 pg/ml Vs PDM, median 41.40 pg/ml; IQR, 41.40 -44.49 pg/ml: p<0.0001) and IL-2 (TB-PDM-median, 95.20 pg/ml; IQR, 95.20 -97.23 pg/ml Vs TB, median 57.12 pg/ml; IQR, 57.12-61.98 pg/ml Vs PDM, median 38.08 pg/ml; IQR, 38.08-41.32 pg/ml: p<0.0001). Additionally, the level of the type 17 cytokine IL-17A was significantly higher in the TB-PDM group (TB-PDM-median, 53.31 pg/ml; IQR, 50.95-55.68 pg/ml Vs TB, median 44.34 pg/ml; IQR, 37.95-45.88 pg/ml Vs PDM, median 30.57 pg/ml; IQR, 30.57-33.41 pg/ml: p<0.0001) than in those with TB alone or PDM alone. The findings also revealed that the levels of both type 1 and type 17 cytokines were higher in individuals with TB compared to those with PDM. Therefore, TB-PDM is characterized by an increase in both type 1 and type 17 cytokines, indicating a more pronounced immune response in individuals with both TB and PDM.

### 3.3 TB-PDM exhibits elevated systemic levels of IL-10

In our investigation into the impact of PDM on type 2 and anti-inflammatory cytokines in individuals with active TB, we assessed the circulating concentrations of these cytokines. Specifically, we analyzed type 2 cytokines (IL-4, IL-5, IL-13) and the anti-inflammatory cytokine IL-10 in groups with TB-PDM, TB alone, and PDM alone. As depicted in **Figure 1B**, there were no significant differences in the levels of type 2 cytokines (IL-4, IL-5, IL-13) between the groups. However, the circulating levels of the anti-inflammatory cytokine IL-10 was notably higher in the TB-PDM group (TB-PDM-median, 62.28 pg/ml; IQR, 56.88 - 67.65 pg/ml Vs TB, median 43.89 pg/ml; IQR, 37.14 – 47.40 pg/ml Vs PDM, median 21.36 pg/ml; IQR, 20.16 – 22.55 pg/ml: p<0.0001) compared to individuals with TB alone or PDM alone. Additionally, IL-10 levels were significantly elevated in the TB group compared to those with PDM alone. Therefore, TB-PDM is characterized by increased levels of the anti-inflammatory cytokine IL-10, suggesting a distinct immunological profile in individuals with both TB and PDM.

### 3.4 TB-PDM presents with heightened systemic levels of other pro-inflammatory cytokines and IL-1 family of cytokines

In our investigation into the impact of PDM on the IL-1 family and additional pro-inflammatory cytokines in individuals with active TB, we analyzed the circulating concentrations of IL-1α, IL-1β, IL-6, IL-12, IL-18, and GM-CSF. Comparing individuals with TB-PDM, TB alone, and PDM alone, we observed significant differences in cytokine levels, as depicted in **Figure 1C**. Specifically, the circulating levels of IL-1α (TB-PDM-median, 121.2 pg/ml; IQR, 110.1- 126.1 pg/ml Vs TB, median 69.80 pg/ml; IQR, 66.83 – 72.72 pg/ml Vs PDM, median 46.53 pg/ml; IQR, 46.53- 48.48 pg/ml: p<0.0001), IL-1β (TB-PDM-median, 71.60 pg/ml; IQR, 57.59- 88.51 pg/ml Vs TB, median 44.96 pg/ml; IQR, 34.16 – 56.64 pg/ml Vs PDM, median 29.84 pg/ml; IQR, 28.08 – 35.78 pg/ml: p<0.0001), IL-6 (TB-PDM-median, 180.6 pg/ml; IQR, 149.5 – 235.9 pg/ml Vs TB, median 59.42 pg/ml; IQR, 46.16 – 84.99 pg/ml Vs PDM, median 38.54 pg/ml; IQR, 35.18 – 41.16 pg/ml: p<0.0001), IL-12 (TB-PDM-median, 79.26 pg/ml; IQR, 76.78 – 81.73 pg/ml Vs TB, median 64.14 pg/ml; IQR, 61.37 – 69.26 pg/ml Vs PDM, median 34.63 pg/ml; IQR, 33.99 – 37.14 pg/ml: p<0.0001), and GM-CSF (TB-PDM-median, 131.7 pg/ml; IQR, 119.71- 152.7 pg/ml Vs TB, median 92.7 pg/ml; IQR, 90.06 – 95.34 pg/ml Vs PDM, median 84.14 pg/ml; IQR, 72.59 – 86.84 pg/ml: p<0.0001) were markedly elevated in individuals with TB-PDM compared to those with TB alone and PDM alone. Similarly, these cytokines were significantly increased in individuals with TB compared to those with PDM alone. These findings indicate that TB-PDM is characterized by heightened systemic levels of the IL-1 family of cytokines and other pro-inflammatory cytokines, suggesting an enhanced inflammatory response in individuals with both TB and PDM.

### 3.5 TB-PDM is associated with elevated systemic levels of CC and CXC chemokines

**Figure 2:**
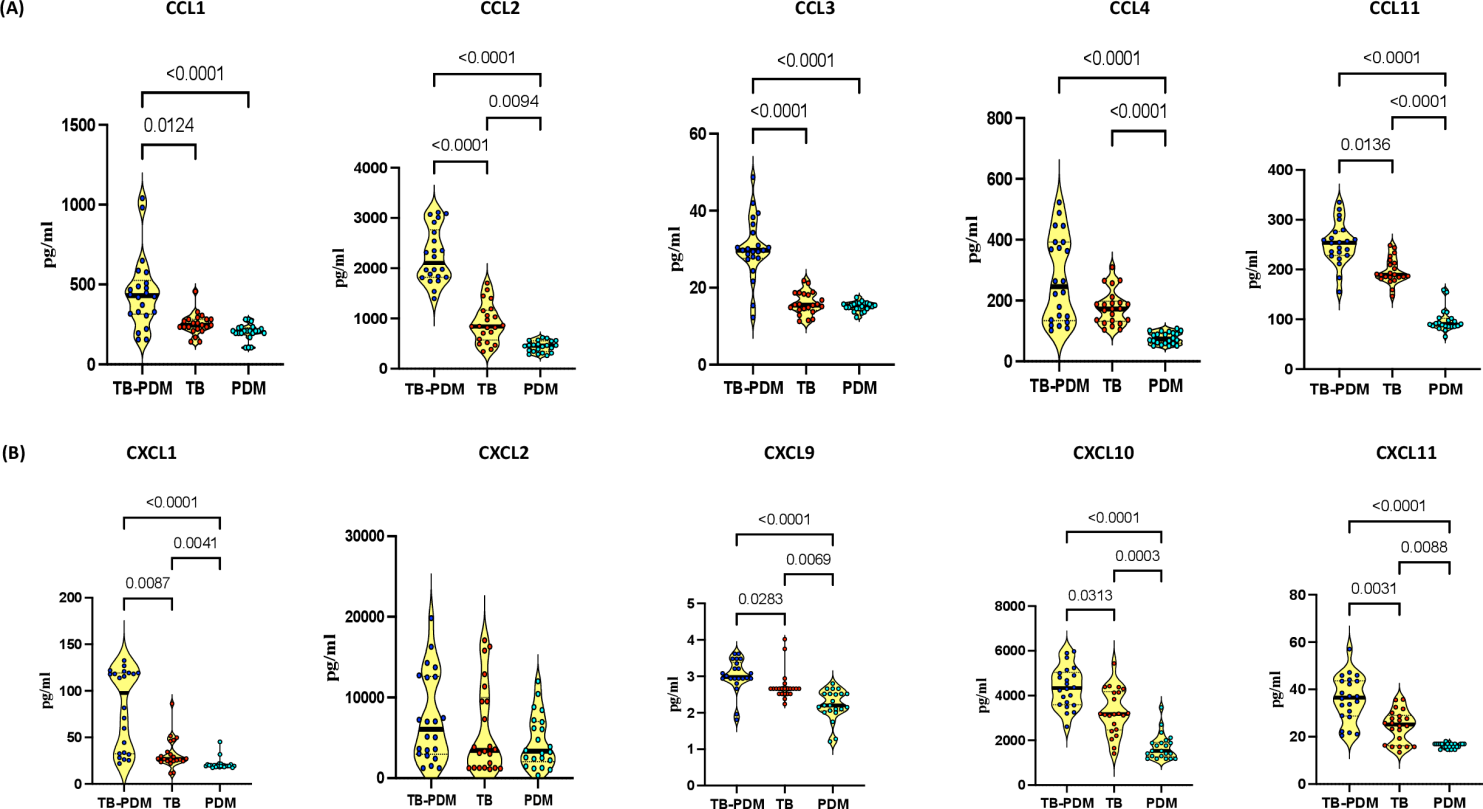
Association of TB-PDM with Increased Levels of CC and CXC Chemokines. Plasma levels of **(A)** CC chemokines (CCL1, CCL2, CCL3, CCL4, and CCL11) and **(B)** CXC chemokines (CXCL1, CXCL9, CXCL10, and CXCL11) were assessed via Bio-Rad, MAGPIX multiplex system in individuals with TB-PDM (n=22), PDM (n=22), and TB (n=22). Data are represented as violin plots, with each circle indicating an individual (Navy Blue – TB-PDM; Maroon – TB; Light Blue – PDM). p-values were determined using the Kruskal-Wallis test with Dunn’s multiple corrections.

In our investigation to assess the impact of PDM on CC and CXC chemokines in individuals with active TB, we analyzed the circulating concentrations of various chemokines including CCL1, CCL2, CCL3, CCL4, CCL11, CXCL1, CXCL2, CXCL9, CXCL10, and CXCL11. Comparing individuals with TB-PDM, TB alone, and PDM alone, we observed notable differences in chemokine levels, as illustrated in **Figure 2**. As shown in **Figure 2A**, CCL2 (TB-PDM-median, 2105 pg/ml; IQR, 1820 - 2766 pg/ml Vs TB, median 839.5 pg/ml; IQR, 568 – 1160 pg/ml Vs PDM, median 458.6 pg/ml; IQR, 337.2 – 554.4 pg/ml: p<0.0001), CCL4 (TB-PDM-median, 245.4 pg/ml; IQR, 133.8 – 392.3 pg/ml Vs TB, median 172.7 pg/ml; IQR, 131.7 – 197.9 pg/ml Vs PDM, median 73.03 pg/ml; IQR, 59.36 – 94.31 pg/ml: p<0.0001), and CCL11 (TB-PDM-median, 253.9 pg/ml; IQR, 228.1 – 276.5 pg/ml Vs TB, median 186.6 pg/ml; IQR, 183.8 – 214.2 pg/ml Vs PDM, median 90.37 pg/ml; IQR, 85.66 – 102.1 pg/ml: p<0.0001) levels were elevated in TB compared to PDM individuals, indicating a distinct chemokine profile associated with TB-PDM. Regarding CXC chemokines, individuals with TB-PDM exhibited significantly higher levels of CXCL1 (TB-PDM-median, 97.79 pg/ml; IQR, 32.38- 119.2 pg/ml Vs TB, median 26.99 pg/ml; IQR, 24.52 – 37.34 pg/ml Vs PDM, median 19.17 pg/ml; IQR, 18.95 – 20.95 pg/ml: p<0.0001), CXCL9 (TB-PDM-median, 2.980 pg/ml; IQR, 2.940 – 3.383 pg/ml Vs TB, median 2.660 pg/ml; IQR, 2.520 – 2.660 pg/ml Vs PDM, median 2.200 pg/ml; IQR, 2.048 – 2.520 pg/ml: p<0.0001), CXCL10 (TB-PDM-median, 4336 pg/ml; IQR, 3588 - 5036 pg/ml Vs TB, median 3176 pg/ml; IQR, 2465 – 4179 pg/ml Vs PDM, median 1529 pg/ml; IQR, 1265 - 1912 pg/ml: p<0.0001), and CXCL11 (TB-PDM-median, 36.58 pg/ml; IQR, 28.75 – 43.64 pg/ml Vs TB, median 25.18 pg/ml; IQR, 16.21 – 29.06 pg/ml Vs PDM, median 16.95 pg/ml; IQR, 15.77 – 16.95 pg/ml: p<0.0001) compared to those with TB alone (**Figure 2B**). Additionally, circulating levels of CXCL1, CXCL9, CXCL10, and CXCL11 were significantly elevated in TB individuals compared to those with PDM alone. However, no significant differences were observed in the level of CXCL2 between the groups. These findings suggest that TB-PDM is characterized by markedly elevated systemic levels of both CC and CXC chemokines.

### 3.6 Examining the relationship between systemic cytokines/chemokines and HbA1c levels

**Figure 3:**
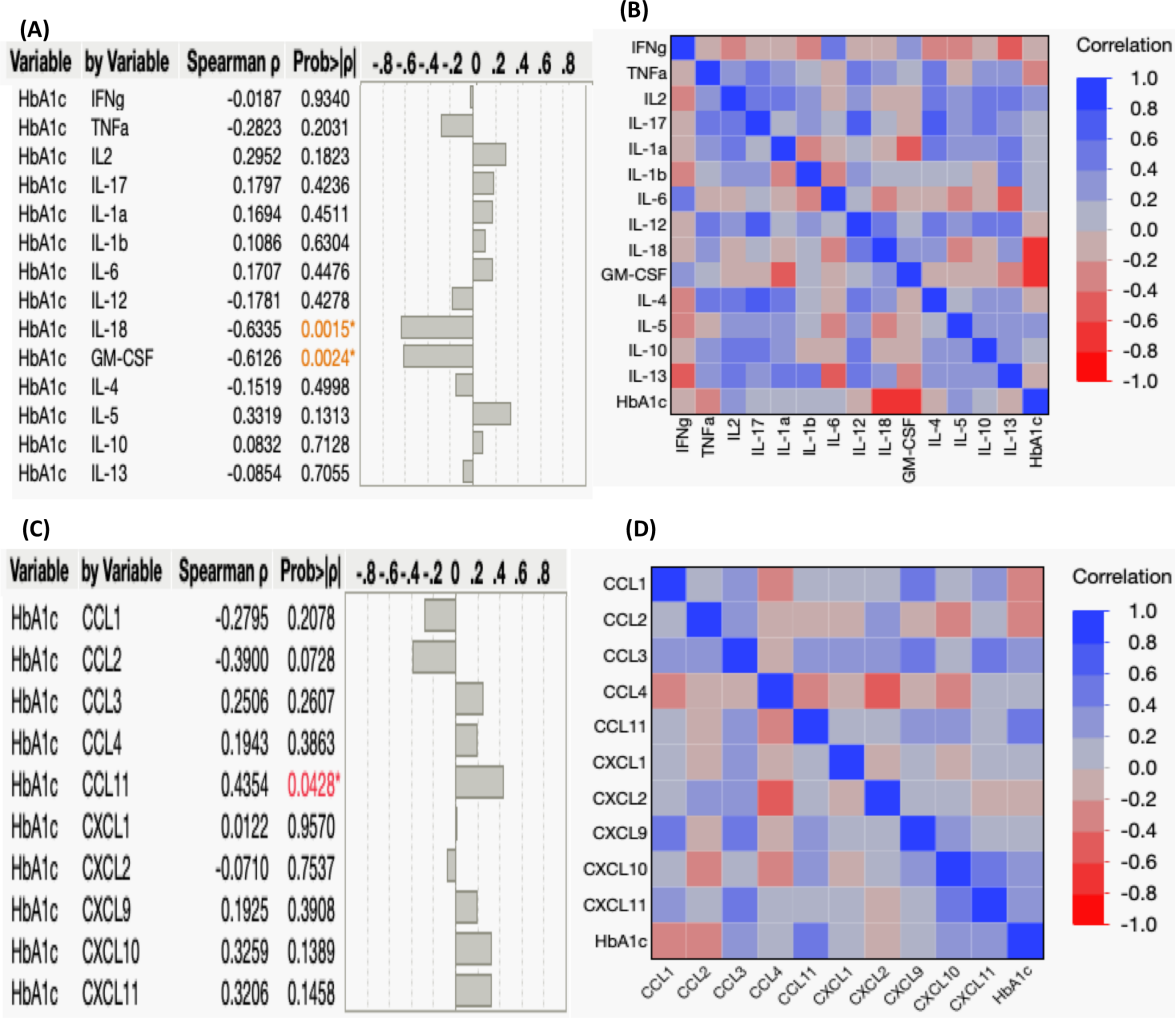
Correlation between Systemic Cytokines, Chemokines, and HbA1c Levels in TB-PDM. **(A)** A multiparametric matrix correlation plot depicts the relationships between plasma cytokines (Type 1, Type 17, Type 2, regulatory, and other pro-inflammatory) and HbA1c levels in TB-PDM patients. **(B)** Spearman’s correlation coefficients are visualized, with blue indicating positive correlations and red indicating negative correlations. **(C)** A multiparametric matrix correlation plot depicting the relationships between plasma CC and CXC chemokines and HbA1c levels in TB-PDM patients. **(D)** Spearman’s correlation coefficients are visualized, with blue indicating positive correlations and red indicating negative correlations.

Our study aimed to elucidate the connection between systemic cytokine and chemokine levels and the degree of glycemic control, as reflected by HbA1c levels, in individuals with TB-PDM. Elevated HbA1c values indicate poor blood sugar control. We assessed the correlation between HbA1c levels (%) and the circulating levels of various cytokines and chemokines. First, regarding cytokines, we observed a negative correlation between HbA1c levels and the systemic levels of IL-18 (r= -0.6335; p=0.0015) and GM-CSF (r= -0.6126; p=0.0024), as depicted in **Figures 3A, B**. However, we did not find significant correlations between HbA1c levels and other systemic cytokines in TB-PDM individuals. Furthermore, we explored the association between systemic chemokine levels and glycemic control in **Figures 3C, D**. We investigated the correlation between HbA1c levels (%) and the levels of CC and CXC chemokines, including CCL1, CCL2, CCL3, CCL4, CCL11, CXCL1, CXCL2, CXCL9, CXCL10, and CXCL11. Among the chemokines, CCL11 alone showed a positive correlation with the levels of HbA1c (r= 0.4354; p=0.0428).

**Figure 4:**
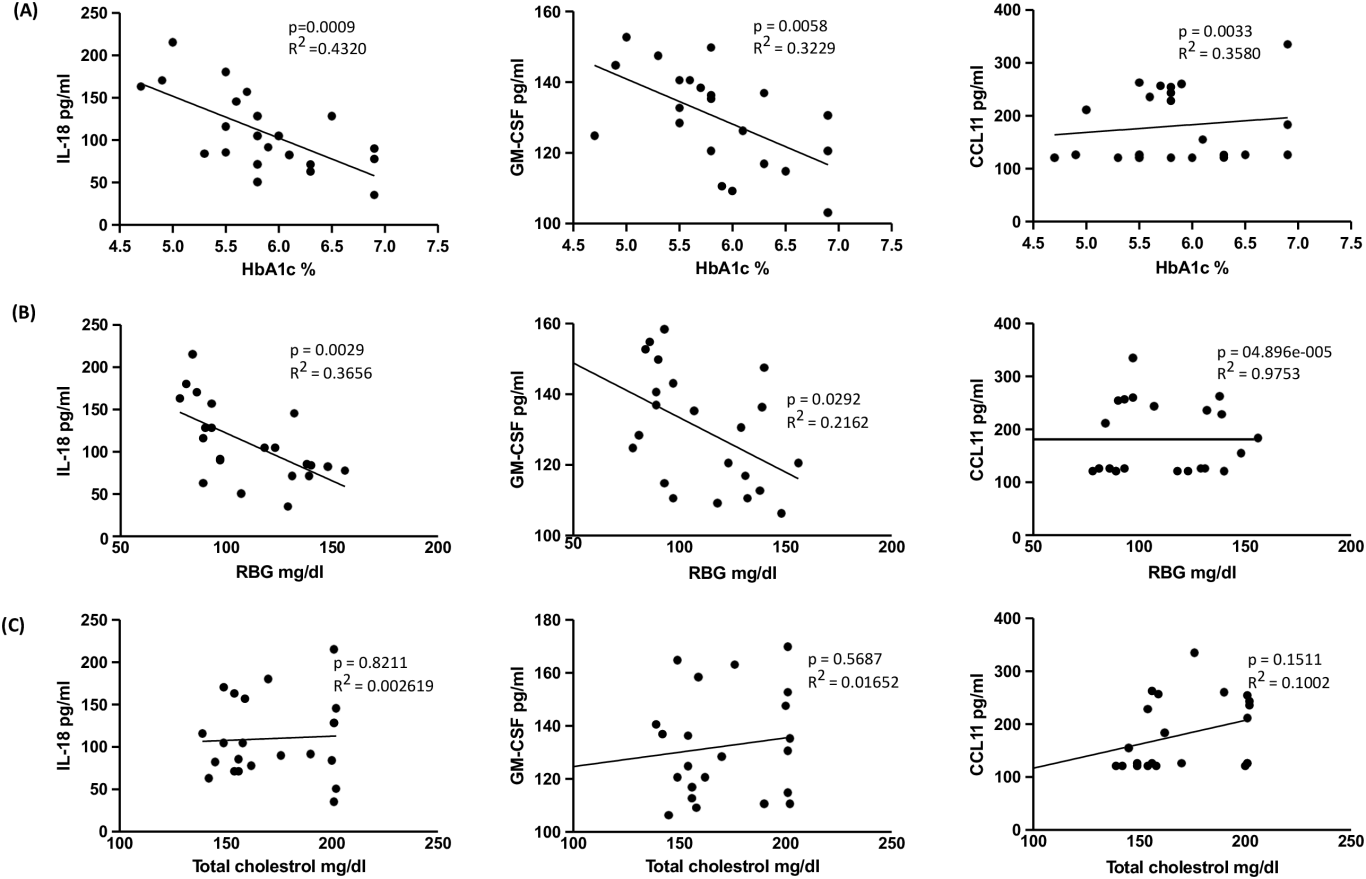
Relationship between IL-18, GM-CSF and CCL11 with HbA1c, RBG and Total cholesterol levels in TB-PDM. **(A)** Linear regression analysis depicting the relationship of cytokines IL-18 and GM-CSF and chemokine CCL11 with HbA1c%. **(B)** Linear regression analysis depicting the relationship of cytokines IL-18 and GM-CSF and chemokine CCL11 with RBG. **(C)** Linear regression analysis depicting the relationship of cytokines IL-18 and GM-CSF and chemokine CCL11 with the levels of Total cholesterol. R^2^ values and p-values are indicated for each relationship, with statistically significant associations (p < 0.05).

The linear regression analysis revealed significant associations between specific cytokines and HbA1c levels. As illustrated in **Figure 4A**, IL-18 (R^2^ = 0.4320; p = 0.0009), GM-CSF (R^2^ = 0.3229; p = 0.0058), and CCL11 (R^2^ = 0.3580; p = 0.0033) demonstrated substantial positive correlations. In **Figure 4B**, IL-18 (R^2^ = 0.3656; p = 0.0029) and GM-CSF (R^2^ = 0.2162; p = 0.0292) also exhibited significant positive relationships with RBG levels, while CCL11 (R^2^ = 04.896e-005; p = 0.9753) did not show a significant association with RBG levels. Conversely, no significant relationship was observed between total cholesterol levels and cytokines or chemokines. Consequently, only specific cytokines and chemokines were found to be associated with HbA1c (%) and RBG levels.

## 4 Discussion

Diabetes mellitus (DM) and its precursor, PDM, are linked to immune system dysfunction, involving alterations in cytokine and chemokine levels, changes in immune cell types and activation status, and increased apoptosis and tissue fibrosis (25). PDM shares characteristics of glucose dysregulation and insulin resistance with DM, potentially affecting susceptibility to TB (10, 26). Although TB patients with PDM exhibit cytokines and chemokines profile akin to type 2 diabetes (T2D), the impact of PDM on TB severity remains unclear. In our study, TB patients with PDM demonstrated elevated levels of various cytokines (IFNγ, TNFα, IL-2, IL-17, IL-1α, IL-1β, IL-6, IL-12, IL-18, and GM-CSF) and chemokines (CCL1, CCL2, CCL3, CCL4, CCL11, CXCL1, CXCL9, CXCL10, and CXCL11). Our data also suggest that PDM is associated with increased bacterial burdens but not disease severity.

Cytokines play a crucial role in TB progression and host defense (27, 28). PDM and metabolic dysfunction are associated with mild inflammation, as evidenced by elevated levels of pro-inflammatory cytokines observed in TB patients with PDM (29). Key cytokines like IFN-γ, TNF-α, IL-17A, IL-1α, IL-1β, IL-18, IL-12, and IL-6 are vital in TB infections (30–33). IFN-γ and TNF-α play crucial roles in *M. tb* infections, IFN-γ, for instance, plays a crucial role in activating macrophages to combat intracellular mycobacteria, while TNF-α contributes to the formation of granulomas, essential for containing mycobacterial growth, while IL-17A mediates memory immune responses and appears to exacerbate inflammation in TB patients with conditions like diabetes, potentially worsening disease severity. IL-1 family cytokines, including IL-1α and IL-1β, are essential for resistance, and IL-18 and IL-12 are vital for immunity. IL-1α and IL-1β initiate and sustain inflammatory responses against mycobacteria, whereas IL-18 enhances IFN-γ production crucial for effective immune responses. IL-6 inhibits disease progression. IL-12 drives Th1 differentiation and IFN-γ production, pivotal in combating TB, and IL-6 has diverse effects including pro-inflammatory responses and B cell activation (30–33). Furthermore, IL-18 and IL-12 are crucial for immunity against *M. tb* infection (34, 35). Elevated systemic pro-inflammatory cytokines, common in T2D, are associated with increased TB risk (11, 12, 34–37). We have also previously shown that LTB with PDM is associated with alterations in cytokine production of NK cells, NKT cells, MAIT cells, and γδ T cells (38, 39). In our study, the TB-PDM group exhibited heightened levels of various cytokines compared to TB or PDM alone. Disease severity and bacterial burden were notably linked to this group, possibly due to chronic low-grade inflammation induced by insulin resistance or dysfunctional adipose tissue (11, 40). Elevated IL-17 levels in TB patients with diabetes may worsen inflammation and pathology, contributing to more severe TB disease in individuals with T2D. (13, 14, 41). The blood transcriptomic profiles of TB patients with pre-diabetes resemble those of TB patients with T2D more closely than those without dysglycemia, indicating that immune responses to *M.tb* are impaired in the early stages of dysglycemia in PDM (10). This is very similar to our previous findings in overt DM and PTB (9). Our data suggest that early stages of dysglycemia may contribute to the pro-inflammatory environment in PDM patients.

IL-1α and IL-1β are critical for TB resistance, as evidenced by studies in mice (31, 42). Elevated IL-10 levels in T2D patients with TB suggest its role in exacerbated immune dysregulation (31, 43–45) Our findings support previous studies, indicating intensified inflammatory reactions influenced by TB-induced immune dysregulation in the TB-PDM group. T2D may worsen TB severity by reducing alveolar macrophage activation via decreased IL-1β, IL-12, and IL-18 release (46, 47). Our findings suggest increased levels of pro-inflammatory cytokines and heightened responses from Th1 and Th17 cells and cytokines in patients with TB-PDM. Addressing cytokine imbalances in TB and PDM individuals could improve treatment outcomes.

In the context of TB and concurrent PDM, inflammation plays a crucial role, with chemokines emerging as key players (48–50). This inflammatory environment can activate cytokine signaling proteins, contributing to insulin resistance. Chemokines are vital for recruiting immune cells to the lung during early infection stages (50, 51). Notably, chemokines like CCL1, CCL2, CCL4, CCL5, CCL11, CXCL8, CXCL10, and CX3CL1 are implicated in T2D pathogenesis, affecting immunoregulation, inflammatory gene induction, and insulin signaling modulation (50). Chemokines act as signaling molecules in inflammation, activating pro-inflammatory mediators and inducing a variety of inflammatory factors. These factors trigger cytokine signaling proteins that impede insulin signaling receptor activation in pancreatic cells, thereby promoting insulin resistance (IR). This sequence of events is implicated in the progression from PDM to T2D (50). However, few studies have explored chemokine levels in TB and T2D comorbidity.

Animal models have shown that abnormalities in specific chemokine synthesis are linked to increased susceptibility to *Mycobacterium tuberculosis* (*M.tb*) infection (52). Animal models of TB and diabetes exhibit exacerbated disease progression, with increased bacterial burden and dysregulated chemokine expression (53). CCR2 is a critical receptor involved in the development of T2D. Adipocytes secrete inactive CCR2, which, when activated, promotes the expression of inflammatory genes and reduces insulin-dependent glucose uptake. Adipocytes also release CCL2, which recruits macrophages to the site of inflammation. These mechanisms contribute significantly to the pathogenesis of T2D (50). CXCL10 plays a crucial role in initiating the destruction of β cells. Additionally, CXCL10 can impair insulin secretion and reduce the viability of β cells. The specific mechanism involves CXCL10-inducing dysfunction in β cells, which has been shown to be elevated in T2D patients (50). Our findings support these, showing elevated CCL2/MCP-1 and CXCL10 levels in the TB-PDM group, indicating increased bacterial burdens associated with dysregulated chemokine expression. Individuals who have both TB and PDM comorbidity exhibit elevated levels of pro-inflammatory cytokines. The interaction between TB and PDM potentially promotes pathology by enhancing the production of cytokines, potentially exacerbating the progression of diabetes mellitus (9).

Further, the linear regression analysis suggests that IL-18 and GM-CSF play significant roles in glucose metabolism as indicated by their associations with HbA1c and RBG levels. CCL11, while associated with HbA1c, did not correlate significantly with RBG levels, highlighting potential differences in its involvement in glucose regulation compared to IL-18 and GM-CSF. Further research into these cytokines’ mechanistic roles and clinical implications could pave the way for targeted therapeutic approaches in managing pre-diabetes and preventing its progression to type 2 diabetes.

Our cross-sectional study lacks the ability to establish definitive cause-and-effect relationships between PDM and TB. Our study also suffers from the limitation of a small sample size. However, our findings suggest that individuals with both TB and PDM have elevated levels of pro-inflammatory cytokines and chemokines compared to those with either TB or PDM alone, potentially exacerbating TB pathogenesis in these patients. In TB-PDM comorbidity, these factors likely synergize, exacerbating inflammation and immune dysregulation, thus complicating disease progression. Understanding these mechanisms is crucial for developing effective strategies to manage TB-PDM comorbidity. Longitudinal studies are needed to determine causation and understand the complex processes underlying the relationship between PDM and TB. This study did not investigate the responses following anti-TB treatment. However, future studies addressing the reversibility of PDM and the impact of anti-TB treatment on diabetes status would provide valuable clinical insights.

## Data Availability

The original contributions presented in the study are included in the article/supplementary material. Further inquiries can be directed to the corresponding author.
